# Pickering Emulsion Biocatalysis with Engineered Living Cells for Degrading Polycarbonate Plastics

**DOI:** 10.1002/smll.202504376

**Published:** 2025-05-24

**Authors:** Shan Wang, Zhimin Gong, René Hübner, Henrik Karring, Changzhu Wu

**Affiliations:** ^1^ Department of Physics Chemistry and Pharmacy University of Southern Denmark Campusvej 55 Odense 5230 Denmark; ^2^ School of Geographic Information and Tourism Chuzhou University Chuzhou 239000 P.R. China; ^3^ Helmholtz‐Zentrum Dresden – Rossendorf (HZDR) Institute of Ion Beam Physics and Materials Research Bautzner Landstrasse 400 01328 Dresden Germany; ^4^ Department of Green Technology University of Southern Denmark Campusvej 55 Odense 5230 Denmark; ^5^ Danish Institute for Advanced Study (DIAS) University of Southern Denmark Campusvej 55 Odense 5230 Denmark

**Keywords:** interfacial catalysis, pickering emulsion, plastic degradation, polymeric coating, whole‐cell

## Abstract

The efficient degradation of plastics remains a pressing environmental challenge due to their inherent resistance to breakdown. While biocatalysis offers a promising approach for sustainable and effective plastic degradation, the inherently low solubility of plastics in aqueous systems severely limits the efficiency of enzymatic reactions. To address this issue, we developed a biocompatible polymer coating strategy to engineer living cell surfaces, enabling the stabilization of Pickering emulsions for over 192 h and significantly enhancing plastic accessibility to biocatalysts. Leveraging this platform, *Escherichia coli* (*E. coli*) cells containing overexpressed *Candida antarctica* Lipase B performed well by dispersing at the emulsion interface of water and toluene, facilitating the efficient biodegradation of polycarbonate (PC) plastics. Under optimized reaction conditions (pH 9, 45 °C), this Pickering emulsion system achieved efficient PC degradation, producing up to 4.5 mm bisphenol A within 72 h—far exceeding the performance of biphasic systems using native *E. coli* cells. The findings highlight the transformative potential of surface‐engineered whole‐cell catalysts in addressing environmental challenges, particularly plastic waste remediation.

## Graphical abstract

A biocompatible coating strategy is developed to engineer living whole cells, enabling the formation of stable Pickering emulsions for over 192 h. Compared to conventional biphasic systems, this platform resulted in improved polycarbonate plastics degradation efficiency, yielding up to 4.5 mm bisphenol A within 72 h, and represents a promising biocatalytic approach for sustainable plastic waste remediation.

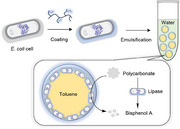

## 1 Introduction

The ever‐present accumulation of plastic waste has become a significant environmental concern, necessitating the development of effective plastic degradation strategies.^[^ ^1^, ^2^, ^3^ ^]^ Conventional approaches, such as physical, chemical, and thermal degradation, often suffer from limitations including high energy demands and the generation of secondary pollutants.^[^ ^4^, ^5^ ^]^ Biodegradation, using biological catalysts such as enzymes or whole cells, has emerged as a promising alternative due to its environmental compatibility and high efficiency.^[^ ^6^, ^7^, ^8^, ^9^ ^]^ However, the biodegradation of plastics is challenged by several factors, including a low adaptability of biocatalysts and the poor solubility of plastics in aqueous systems, which hinder effective substrate accessibility.^[^ ^10^, ^11^, ^12^, ^13^, ^14^ ^]^ One potential solution to this issue is dissolving plastics in organic solvents, which can greatly improve their solubility and subsequently enhance their reaction rates with biocatalysts.^[^ ^15^, ^16^ ^]^ This strategy lays the groundwork for more efficient plastic degradation systems, among which interfacial biocatalysis has shown significant potential owing to its capacity to significantly enhance the interfacial area between the substrate and the biocatalyst.^[^ ^17^, ^18^, ^19^ ^]^

Interfacial biocatalysis operates at phase boundaries, such as oil‐water or solid–liquid interfaces, providing enhanced substrate availability and catalytic efficiency compared to conventional two‐phase reaction systems.^[^ ^20^, ^21^, ^22^ ^]^ This approach has demonstrated considerable success in fields like fine chemical synthesis and pharmaceutical production.^[^ ^23^, ^24^, ^25^, ^26^, ^27^ ^]^ Among interfacial biocatalysis systems, whole‐cell biocatalysis offers unique advantages, including ease of recovery, elimination of enzyme purification steps, and cost‐effectiveness.^[^ ^28^, ^29^, ^30^ ^]^ These benefits have led to its increasing application in interfacial biocatalysis.^[^ ^31^, ^32^, ^33^, ^34^, ^35^, ^36^ ^]^ For example, our group previously demonstrated the functionalization of *Escherichia coli* (*E. coli*) cell surfaces with dopamine coatings to create artificial spores, and these cells with artificial spores effectively stabilized aqueous‐organic emulsions, enabling efficient interfacial biocatalysis.^[^ ^37^ ^]^ This approach has been successfully applied to a wide range of reactions, from single‐enzyme processes to multi‐enzyme cascades, and even chemoenzymatic cascade reactions within established Pickering emulsions with living whole cells.^[^ ^37^, ^38^ ^]^ Surprisingly, however, utilizing whole‐cell interfacial biocatalysis for plastic degradation is still underdeveloped. This is mainly due to several difficulties. One key issue is the hydrophilic nature of cell surfaces, which makes it hard to stabilize emulsions and leads to lower catalytic efficiency.^[^ ^39^, ^40^ ^]^ Moreover, the chemical stability of plastics greatly slows down the degradation process.^[^ ^41^, ^42^ ^]^ This problem is further worsened by the lack of long‐term stability in current emulsion systems based on whole cells, which is essential for continuous catalytic degradation.^[^ ^43^, ^44^ ^]^ Thus, it is urgently needed to develop a simple and effective strategy to engineer live cells, enhancing their amphiphilicity for long‐time stable emulsions while preserving cellular viability and functionality for expanding the application in the degradation of plastic waste.

**Figure 1:**
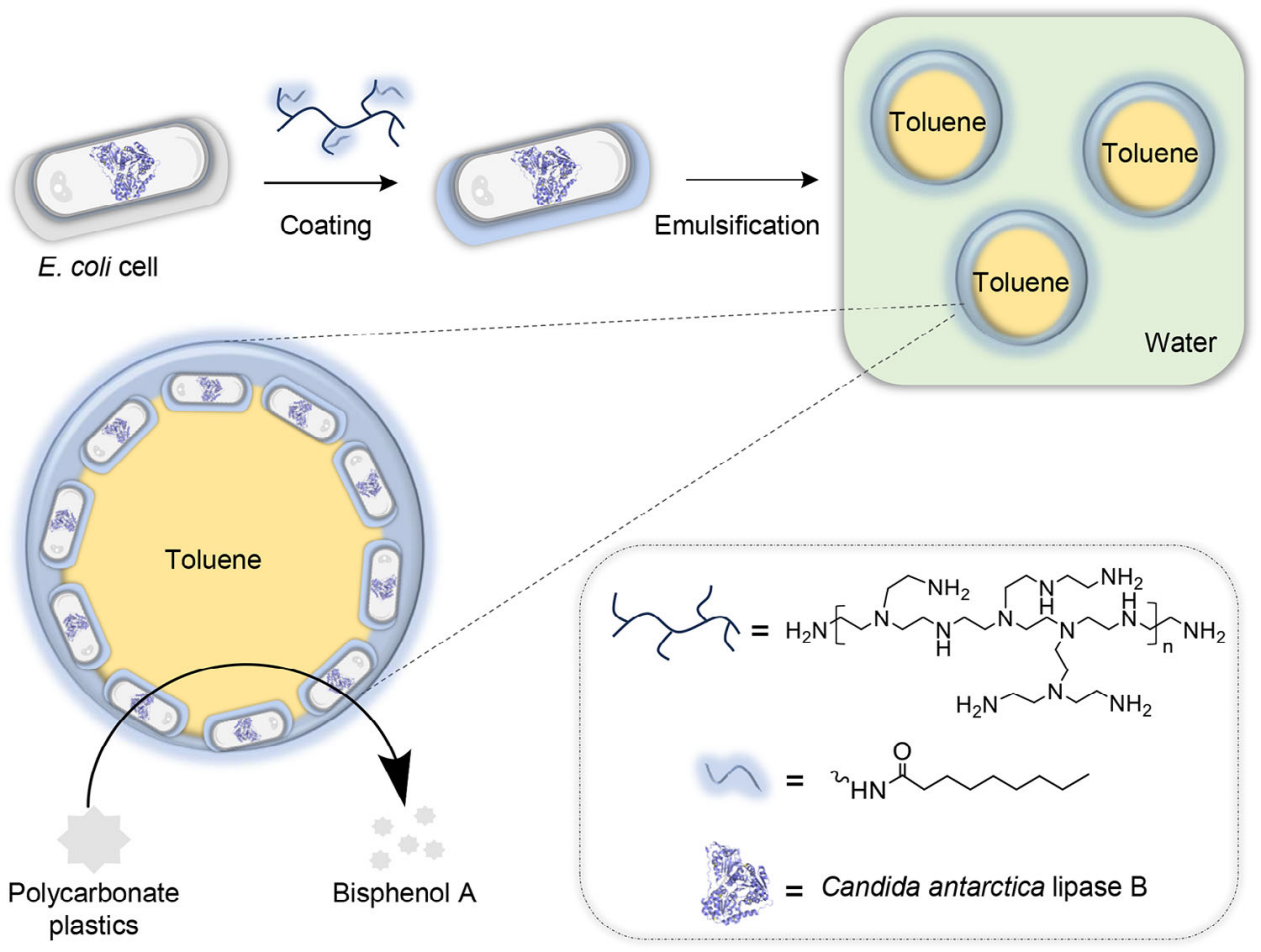
Schematic demonstration for engineering live cells for Pickering interfacial biocatalysis achieving efficient polycarbonate biodegradation.

In this work, we present a robust polymer coating approach to engineer living cell surfaces for efficient interfacial biocatalysis, achieving notable polycarbonate (PC) plastics biodegradation in Pickering emulsions (**Figure** **1**). To achieve this, we designed polyethylenimine polymers modified with hydrophobic alkyl chains to attach to the surfaces of *E. coli* cells via charge–charge interactions. Furthermore, we demonstrate that this biocompatible coating strategy exerts minimal impact on cellular growth and metabolic activity. By leveraging the polymeric coating, we successfully modified the surface of *E. coli* cells to achieve amphiphilicity through effective hydrophobic functionalization. The resulting polymer‐coated *E. coli* cells exhibited noticeable emulsion stabilization capabilities, maintaining emulsion stability for an unprecedented duration exceeding 192 h. Building on this platform, we successfully expressed *Candida antarctica* Lipase B (CalB) in polymer‐coated *E. coli* cells to enable the biodegradation of PC plastics in Pickering emulsion. Therefore, our study introduces a robust polymer coating strategy to engineer the amphiphilicity of live cell surfaces, tailored for long‐time stable emulsion formation. This approach not only accelerates the efficient biodegradation of PC plastics but also paves the way for transformative advancements in synthetic chemistry, plastic degradation, and biocatalysis.

## 2 Results and Discussion

### 2.1 Hydrophobic Modification of *E. coli* Cell Surface

**Figure 2:**
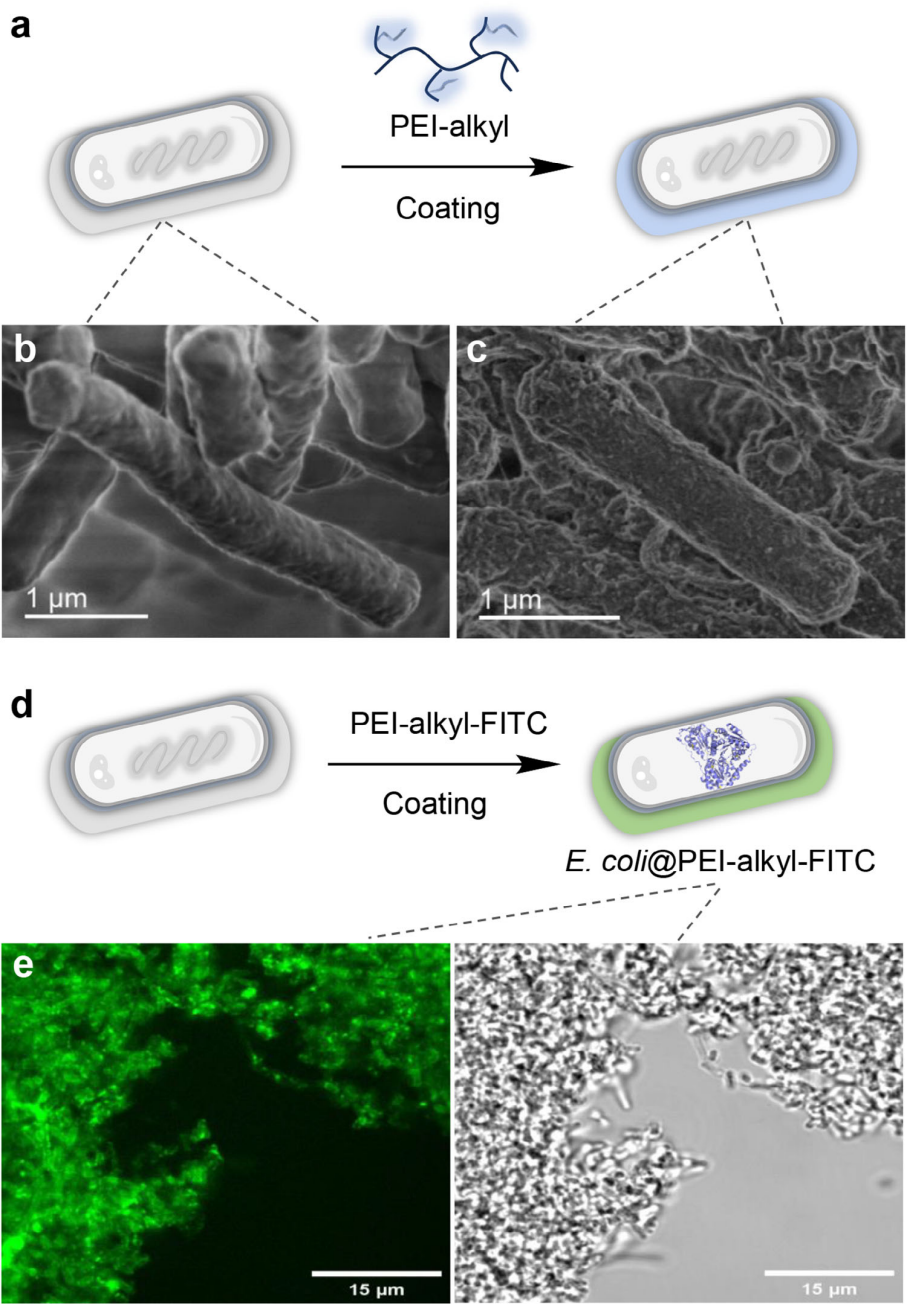
Characterization of polymer coating on *E. coli* cell surface. a) Scheme of the hydrophobic modification on *E. coli* cell surface. SEM images of native *E. coli* cells b) and *E. coli*@alkyl cells c). d) Schematic representation of the polymeric coating incorporating dye, PEI‐alkyl‐FITC. e) Fluorescence microscopy image (left) and corresponding optical microscopy image (right) of *E. coli*@alkyl‐FITC.

To achieve interfacial biocatalysis for living *E. coli* cells in two‐phase systems, surface hydrophobic modification presents a promising strategy to endow *E. coli* cells with amphiphilic properties for stabilizing Pickering emulsions.^[^ ^37^, ^45^ ^]^ Here, a simple two‐step polymer coating process was developed to functionalize *E. coli* cells, thereby creating a robust interfacial whole‐cell catalysis (**Figure** **2**a). In the first‐step, polyethyleneimine (PEI) was alkylated using nonanoyl chloride (Scheme S1, Supporting Information), yielding alkylated PEI (denoted as PEI‐alkyl), confirmed by the appearance of alkyl chain chemical shifts (0.6–1.5 ppm) in proton nuclear magnetic resonance (^1^H NMR) spectra (Figure S1, Supporting Information).^[^ ^46^ ^]^ In the second step, the positively charged PEI‐alkyl interacted effectively with the negatively charged native *E. coli* cells, forming a stable surface coating. The PEI‐alkyl‐coated cells, referred to as *E. coli*@alkyl, were obtained after thorough washing steps to remove unbound PEI‐alkyl.

To validate the successful deposition of the PEI‐alkyl polymer coating on the cell surface, scanning electron microscopy (SEM) was employed. The analysis revealed distinct morphological differences between native and polymer‐coated cells. As shown in Figure 2b, native *E. coli* cells displayed smooth, unaltered membranes, whereas *E. coli*@alkyl cells exhibited rougher surfaces, indicative of a dense PEI‐alkyl polymer layer (Figure 2c). Furthermore, the presence of PEI‐alkyl on the *E. coli* surface was confirmed via fluorescence microscopy by partially modifying PEI‐alkyl with the fluorescein isothiocyanate (Scheme S2, Supporting Information), denoted as PEI‐alkyl‐FITC, followed by coating on *E. coli* cells to obtain dye‐coated *E. coli* cells with PEI‐alkyl‐FITC (*E. coli*@alkyl‐FITC, Figure 2d). Notably, these cells retained strong fluorescence signals even after extensive washing, demonstrating the stability and robustness of the PEI‐alkyl polymeric coating. Additionally, optical microscopy revealed significant cell aggregation of the coated *E. coli* cells, likely due to random interactions mediated by the charged PEI‐alkyl polymers (Figure 2e).^[^ ^38^ ^]^ In contrast, no such aggregation was observed in native cells. Collectively, these results confirm the successful formation of a robust PEI‐alkyl polymer coating on the *E. coli* cell surface.

These results highlight the successful modification of live *E. coli* cells with alkyl chain polymer coatings, paving the way for their application in interfacial catalysis.

### 2.2 Viability and Proliferation of *E. coli*@alkyl

**Figure 3:**
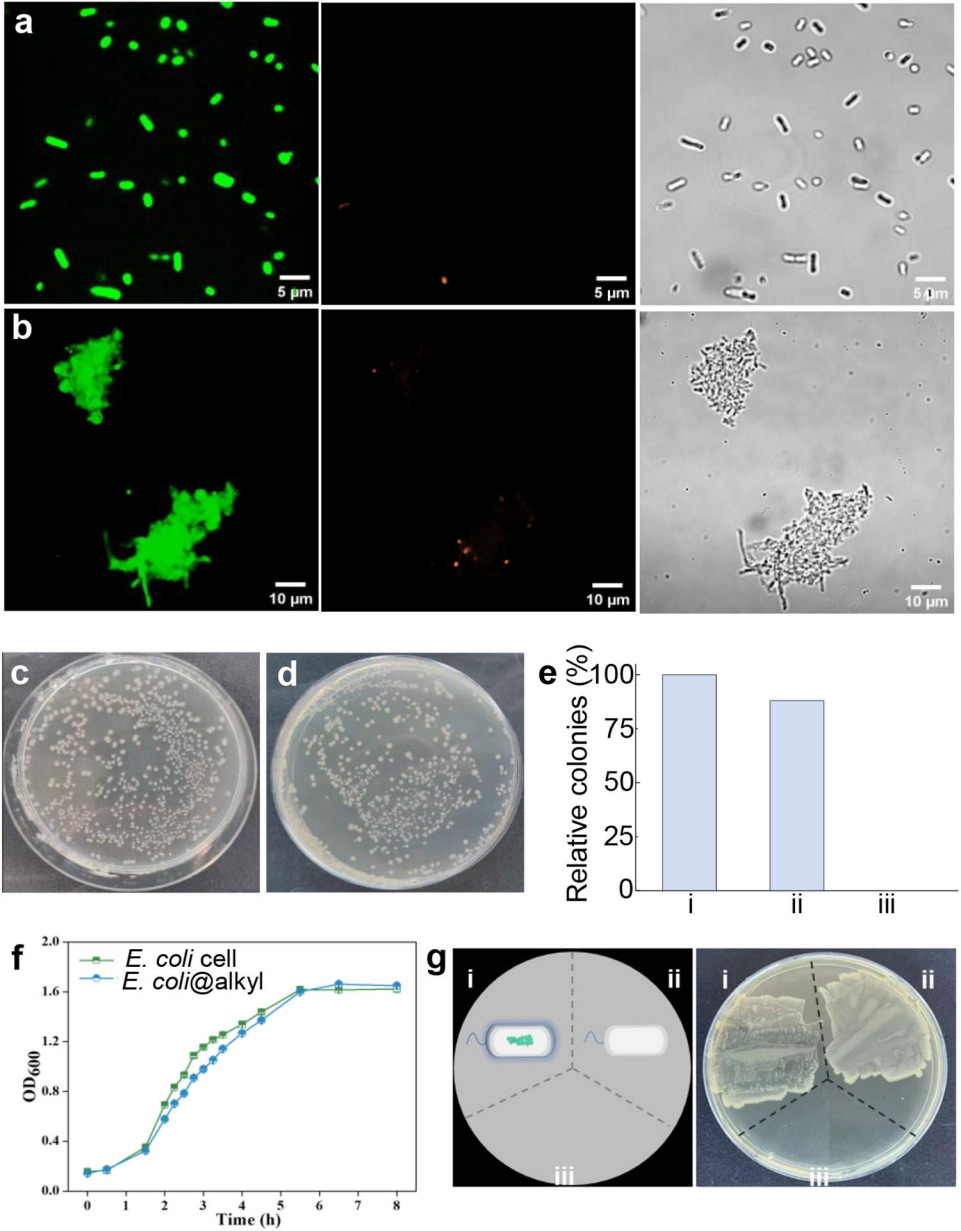
Biocompatibility of the PEI‐alkyl polymer coating. Live/dead assay images for original native *E. coli* cells a) and *E. coli*@alkyl cells b): live cells (left), dead cells (middle), and optical microscopy images (right), respectively. Plating assay of native cells c) and *E. coli*@alkyl cells d). Relative colonies of native *E. coli* cells i), *E. coli*@alkyl ii), and dead cells iii) e). Growth curve of native *E. coli* cells and *E. coli*@alkyl cells f). Values are expressed as mean ± standard deviation (s.d.), calculated from three independent replicates (*n* = 3); error bars indicate the s.d. Plating assay for *E. coli*@alkyl i), native *E. coli* cells ii), and dead *E. coli* cells iii), respectively (g).

The successful preparation of PEI‐alkyl polymer coatings on the cell surface prompted us to investigate their potential impact on cell viability. A live/dead assay was conducted using SYTO9 (green) and propidium iodide (red), which selectively stain live and dead cells, respectively.^[^ ^47^ ^]^ This assay was performed on both native *E. coli* cells and *E. coli*@alkyl cells. As shown in **Figure** **3**a, native cells exhibited uniform green fluorescence, indicative of 100% viability. In contrast, *E. coli*@alkyl cells displayed a small proportion of red‐stained dead cells, with an overall viability exceeding 90% (Figure 3b). Furthermore, the coated cells demonstrated noticeable clumping due to the PEI‐alkyl polymer, a distinct difference from the uniform dispersion observed in native cells. This aggregation, likely attributed to random interactions between PEI‐alkyl polymer and *E. coli* cell surfaces, highlights the unique behavior of coated cells compared to uncoated native *E. coli* cells.

To further assess the impact of PEI‐alkyl polymer coating on *E. coli* viability and metabolic activity, we systematically evaluated the growth and reproduction of cells under both solid and liquid cultivation conditions. Growth on Luria–Bertani (LB) agar plates (Figure 3c–e,g) and in LB liquid medium (Figure 3f) revealed no noticeable differences in growth patterns or colony formation between native *E. coli* and *E. coli*@alkyl. Notably, the coated cells retained over 90% of their colony‐forming ability compared to native cells, highlighting the preservation of cellular viability and metabolic functionality (Figure 3e). These results confirm the biocompatibility of the PEI‐alkyl polymer coating process and its negligible impact on the functions of *E. coli*. Importantly, the fundamental ability of the cells to proliferate remains intact, demonstrating that the coating process minimally interferes with their biological activities (Figure 3f,g).

The results collectively demonstrate that the PEI‐alkyl polymer coating is a gentle process, allowing cell division while supporting the creation of hybrid systems that integrate biological and synthetic materials, highlighting its significant potential for industrial applications.

### 2.3 Emulsion Formation by *E. coli*@alkyl

A critical factor for achieving interfacial biodegradation is ensuring the cells have the appropriate surface amphiphilicity for emulsion formation.^[^ ^48^, ^49^ ^]^ The successful coating with PEI‐alkyl polymers strengthens our confidence in employing *E. coli*@alkyl as an efficient stabilizer for emulsions in interface biocatalysis.

To optimize the cell surface properties, the PEI‐alkyl polymer coating was fine‐tuned by adjusting the polymer concentration from 10 to 20, 30, and 40 mg mL^−1^ with the *E. coli* cell OD_600_ of 2.0, resulting in a series of hydrophobized cell surfaces. Emulsions were then prepared by mixing these coated cells with a biphasic mixture of toluene and water, and stability tests revealed that PEI‐alkyl‐coated cells effectively stabilized the emulsions even after 192 h, demonstrating the introduction of PEI‐alkyl significantly enhances the amphiphilicity of the *E. coli* cell surface. Furthermore, excessive PEI‐alkyl coating, such as 40 mg mL^−1^, led to a reduction in emulsion stability, which may be attributed to excessive modification on the cells caused by the PEI‐alkyl polymers (Figure S2, Supporting Information). Based on these findings, 20 mg mL^−1^ of PEI‐alkyl were identified as the optimal coating concentration and were used in subsequent investigations.

**Figure 4:**
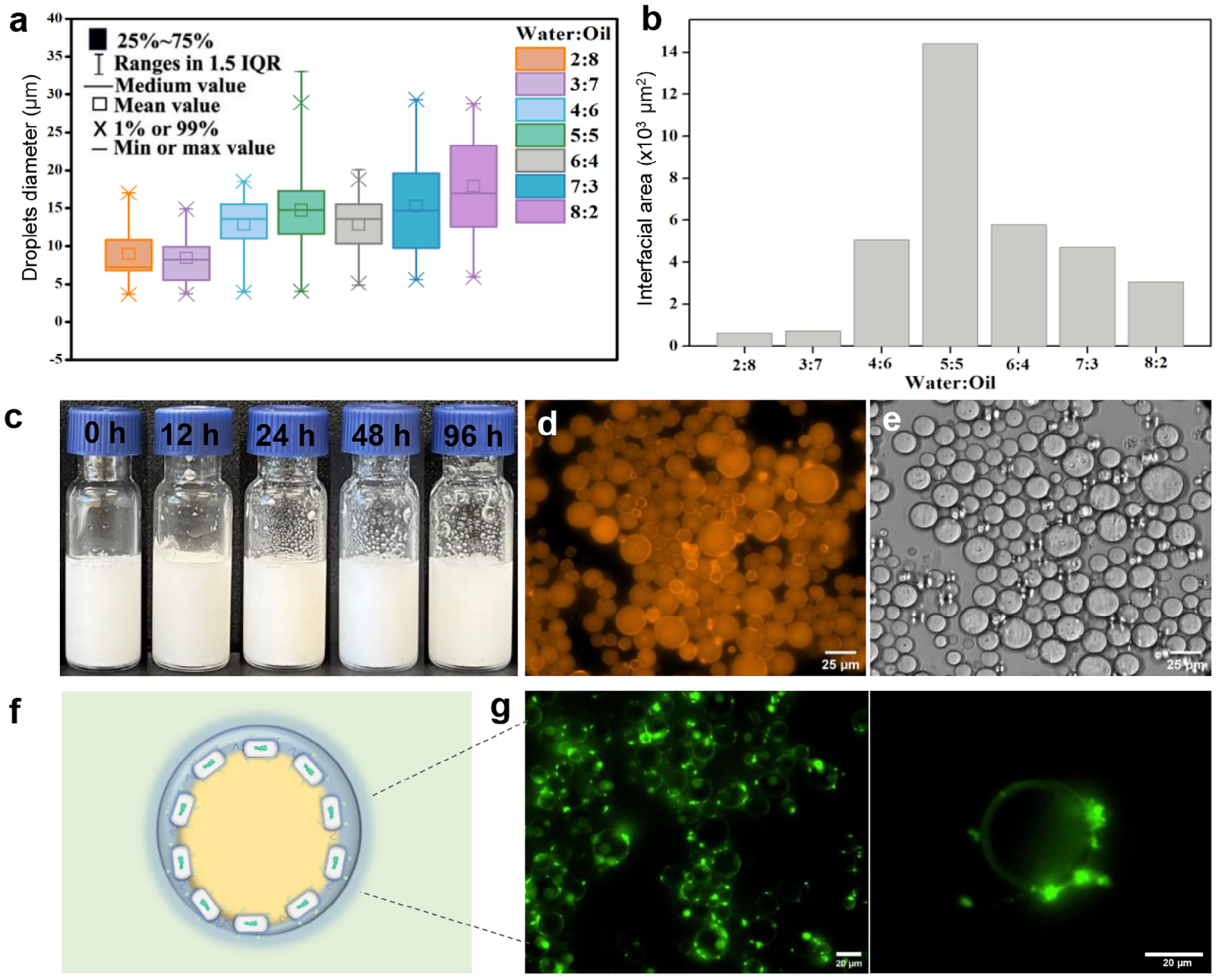
Characterization of emulsions. Box plots of droplet diameters for different water‐to‐oil ratios a). Interfacial areas at different water‐to‐oil ratios b). Emulsion appearance at different times (c). Fluorescence microscopy d) and optical microscopy images e) of the emulsion generated at a 5:5 water‐to‐oil ratio. Scheme of emulsion droplet stabilized by *E coli*(GFP)@alkyl cells f). Fluorescence images of an emulsion stabilized by *E coli*(GFP)@alkyl at different magnifications g).

The stability of the emulsions formed by *E. coli*@alkyl was found to be highly dependent on the water‐to‐oil (W:O) ratio.^[^ ^17^, ^50^ ^]^ To investigate this, emulsions prepared under varying W:O ratios (2:8 to 8:2) were analyzed, and it revealed that emulsions with lower W:O ratios (2:8 to 4:6) exhibited suboptimal volume and dispersibility, likely due to the hydrophilic nature of *E. coli* cells, which impairs effective emulsion formation when the oil phase predominates. In contrast, the 5:5 ratio produced the most stable emulsion, with decent volume and dispersibility. Microscopic analysis provided further insights into the emulsions' characteristics, including size and type. Visible‐light microscopy tests revealed typical emulsion droplet sizes ranging from 5 to 30 µm, while the emulsion type was determined using Nile red, an organic‐solvent‐soluble dye (**Figure** **4**a; Figure S3, S4, Supporting Information).^[^ ^51^ ^]^ Fluorescence microscopy images showed that oil‐in‐water (O/W) emulsions were consistently formed across all W:O ratios, aligning with the hydrophilic nature of *E. coli*@alkyl, which favors O/W emulsion formation.

A further analysis of emulsion photographs revealed that the 5:5 W:O ratio generated the largest interfacial area, outperforming emulsions formed with other ratios (Figure 4b). These results underscore the notable stability and interface accessibility of emulsions at this ratio, highlighting its potential for achieving efficient interfacial biocatalysis.

Under the optimal polymer coating concentration and W:O ratio, the emulsion remained stable for over 96 h (Figure 4c), and fluorescence microscopy images confirmed the characteristic formation of an O/W emulsion (Figure 4d,e). To gain deeper insight into the cell distribution within the emulsions, we prepared emulsions using *E. coli*@alkyl cells overexpressing green fluorescent protein (*E. coli*(GFP)@alkyl) (Figure 4f). The results revealed that the fluorescent cells were predominantly located on the emulsion surface (Figure 4g), confirming that the emulsions were effectively stabilized by *E. coli* cells coated with PEI‐alkyl polymer. As such, this emulsion preparation condition was selected for subsequent interfacial biocatalysis investigations.

### 2.4 PC Biodegradation in Pickering Emulsion

**Figure 5:**
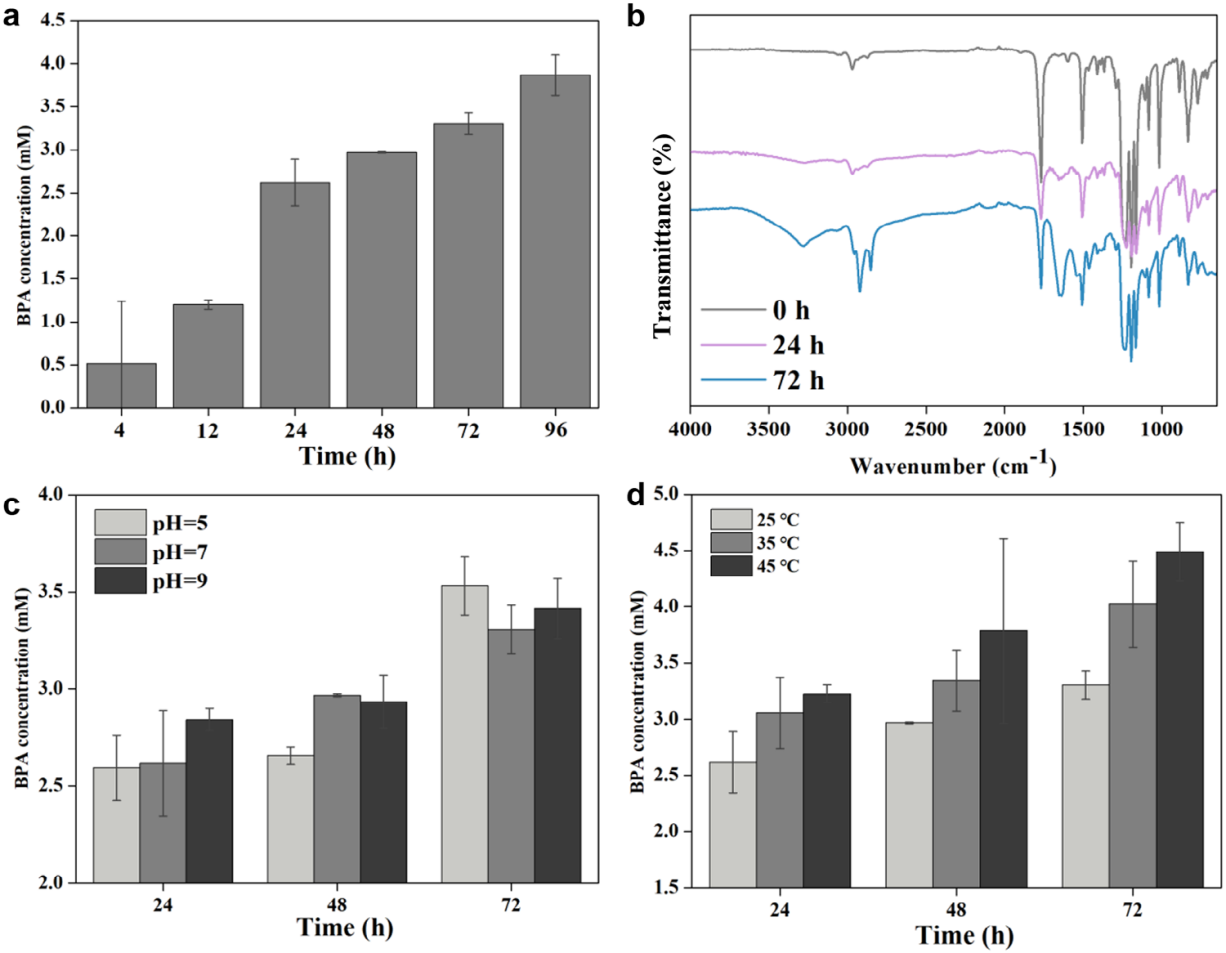
PC biodegradation in Pickering emulsion. Bisphenol A concentration at different reaction times a). FTIR spectra of the crude PC biodegradation product at different reaction times b). The pH c) and temperature d) influence on the biodegradation of PC. Experiments were performed at the conditions of *E. coli* cells with OD_600_ = 2.0, 75 mg mL^−1^ of polycarbonate, and a water: toluene ratio of 1:1. Data in (a,c,d) are expressed as mean ± standard deviation (s.d.), calculated from three independent replicates (*n* = 3); error bars indicate the s.d.

The successful preparation of stable emulsions encouraged us to further explore their application in plastic biodegradation. To this end, polycarbonate, a widely used plastic, was selected as the target substrate.^[^ ^52^, ^53^ ^]^ Prior to coating with the PEI‐alkyl polymer, CalB was successfully expressed within the *E. coli* cells for interfacial biocatalytic degradation of PC. Subsequently, PEI‐alkyl polymer coated *E. coli* cells with CalB (*E. coli*(CalB)@alkyl) were dispersed in 0.5 mL KPi buffer, followed by adding 0.5 mL toluene containing PC to form a stable Pickering emulsion with shaking. As is well known, PC can be enzymatically hydrolyzed by CalB, ultimately degrading into the small molecular compound bisphenol A (BPA).^[^ ^8^, ^54^, ^55^ ^]^ So, we determined the concentration of BPA at different reaction times through withdrawing 20 µL toluene for gas chromatography analysis and observed a gradual increase of BPA content (**Figure** **5**a). Specifically, after 24 h, a significant accumulation of 2.5 mm BPA was detected, and with prolonged reaction time, the BPA concentration further increased, reaching ≈4 mm at 96 h. In comparison, for a biphasic medium with uncoated cells, no detectable BPA was observed even after 96 h. This result clearly demonstrates the enhanced biodegradation capability enabled by the *E. coli*@alkyl‐tailored Pickering emulsion system. The amphiphilicity of *E. coli*(CalB)@alkyl facilitated its localization at the toluene/water interface and promoted the larger interfacial areas between the toluene and water phases, thereby minimizing the mass transfer resistance and contributing to the system's high PC degradation efficiency (Figure S5, Supporting Information). In order to get insight into the PC degradation, the crude degradation products of PC in the Pickering emulsion were collected at different reaction times, and all solvents were removed by evaporation and drying. The samples were further analyzed using Fourier‐transform infrared spectroscopy (FTIR). The results showed the appearance of a distinct new peak at 3350 cm⁻¹, corresponding to the O–H stretching vibration, which was absent at the initial time point.^[^ ^56^ ^]^ This peak is attributed to the formation of BPA as a hydrolysis product of PC by *E. coli*(CalB)@alkyl.^[^ ^57^ ^]^ Moreover, as the reaction time increased, the intensity of this peak became more pronounced (Figure 5b), consistent with the GC results showing a corresponding increase in BPA concentration. Taken together, we have shown that chemically engineering *E. coli* cells surface with amphiphilic polymers could act as an attractive approach for interfacial PC biodegradation.

Furthermore, pH is a critical factor influencing the CalB‐mediated degradation of PC.^[^ ^58^ ^]^ To investigate this, we performed experiments across a pH of 5, 7, and 9. As depicted in Figure 5c, the BPA yield from PC degradation showed minimal differences over this pH range. At pH 9, the BPA concentration reached 2.8 mm after 24 h and increased to 3.4 mm at 72 h. Similarly, at pH 5, the BPA concentration was 2.6 mm at 24 h and rose to 3.6 mm at 72 h. Since pH significantly affects emulsion stability, and the stability of the emulsion directly impacts the interfacial area, it consequently influences the efficiency of PC degradation. As reported, Pickering emulsions demonstrate enhanced stability under alkaline conditions.^[^ ^18^, ^22^ ^]^ Therefore, it is recommended that PC degradation using *E. coli*(CalB)@alkyl in Pickering emulsions is conducted under relatively alkaline conditions to maximize efficiency.

In addition to pH, the temperature also has a strong impact on the CalB‐catalyzed reactions in the Pickering emulsions.^[^ ^18^ ^]^ The result showed a significant increase in BPA concentration produced by PC degradation with rising temperature (Figure 5d). At 24 h, increasing the reaction temperature from 25 to 45 °C led to an increase in BPA concentration from 2.6 to 3.2 mm. When extending the reaction time to 72 h, it showed a similar trend, with the BPA concentration at 45 °C reaching 4.5 mm, compared to 3.3 mm at 25 °C. These results indicate that raising the reaction temperature significantly enhances the whole‐cell CalB‐catalyzed degradation rate of PC in Pickering emulsions. However, considering that excessively high temperatures could compromise the stability of the emulsions, we observed a noticeable decrease in emulsion stability when the temperature exceeded 50 °C. Therefore, we refrained from further increasing the temperature. What's more, under optimized conditions, *E. coli*(CalB)@alkyl could be recycled up to four times while retaining over 80% catalytic activity, demonstrating its robustness and reusability (Figure S6, Supporting Information).

Consequently, we demonstrated that the optimal conditions for PC biodegradation in Pickering emulsions were achieved at pH 9 and reaction temperature of 45 °C. This study provides a simple and efficient method for interfacial biodegradation of polycarbonate plastics and offers a new engineering strategy for plastic‐efficient biodegradation.

## 3 Conclusion

In conclusion, we have successfully engineered live *E. coli* cell surfaces to stabilize emulsions, utilizing them in combination with CalB to effectively biodegrade polycarbonate plastics in long‐time stable Pickering emulsions. Under optimized conditions, we can achieve the efficient degradation of polycarbonate plastics into the small molecule compound, bisphenol A. These findings underscore the significant potential of surface‐engineered whole‐cell catalysts in addressing critical environmental challenges, including plastic waste management. By integrating advancements in polymer coating and interfacial biocatalysis, this study establishes a versatile platform for sustainable bioprocessing. Looking ahead, this approach offers valuable opportunities for the biodegradation of diverse plastic types in Pickering emulsions, promising transformative applications in environmental remediation, synthetic chemistry, and industrial biotechnology.

## Conflict of Interest

The authors declare no conflict of interest.

## Supporting information



Supporting Information

## Data Availability

The data that support the findings of this study are available in the supplementary material of this article.

## References

[1] Y. He , X. Deng , L. Jiang , L. Hao , Y. Shi , M. Lyu , L. Zhang , S. Wang , Sci. Total Environ. 2024, 906, 167850. 37844647 10.1016/j.scitotenv.2023.167850

[2] K. S. Khoo , L. Y. Ho , H. R. Lim , H. Y. Leong , K. W. Chew , J. Hazard. Mater. 2021, 417, 126108. 34020352 10.1016/j.jhazmat.2021.126108 PMC9759681

[3] X. F. Wei , W. Yang , M. S. Hedenqvist , Nat. Commun. 2024, 15, 2052. 38448423 10.1038/s41467-024-46127-9 PMC10917744

[4] F. Zhang , Y. Zhao , D. Wang , M. Yan , J. Zhang , P. Zhang , T. Ding , L. Chen , C. Chen , J. Cleaner Prod. 2021, 282, 124523.

[5] K. Zheng , Y. Wu , Z. Hu , S. Wang , X. Jiao , J. Zhu , Y. Sun , Y. Xie , Chem. Soc. Rev. 2023, 52, 8. 36468343 10.1039/d2cs00688j

[6] J. Singh Jadaun , S. Bansal , A. Sonthalia , A. K. Rai , S. P. Singh , Bioresour. Technol. 2022, 347, 126697. 35026422 10.1016/j.biortech.2022.126697

[7] J. D. Gu , Environ. Sci. Pollut. Res. 2021, 28, 1278. 10.1007/s11356-020-11501-9 33190206

[8] V. Tournier , S. Duquesne , F. Guillamot , H. Cramail , D. Taton , A. Marty , I. André , Chem. Rev. 2023, 123, 5612. 36916764 10.1021/acs.chemrev.2c00644

[9] H. Lu , D. J. Diaz , N. J. Czarnecki , C. Zhu , W. Kim , R. Shroff , D. J. Acosta , B. R. Alexander , H. O. Cole , Y. Zhang , N. A. Lynd , A. D. Ellington , H. S. Alper , Nature 2022, 604, 662. 35478237 10.1038/s41586-022-04599-z

[10] A. R. Bergeson , A. J. Silvera , H. S. Alper , Nat. Commun. 2024, 15, 4715. 38830860 10.1038/s41467-024-49146-8 PMC11148140

[11] F. Nourabi , S. Allahyari , N. Rahemi , Y. K. Mishra , J. Environ. Chem. Eng. 2024, 12, 113818.

[12] T. P. Haider , C. Völker , J. Kramm , K. Landfester , F. R. Wurm , Angew. Chem., Int. Ed. 2019, 58, 50. 10.1002/anie.201805766 29972726

[13] M. Zandieh , E. Griffiths , A. Waldie , S. Li , J. Honek , F. Rezanezhad , P. Van Cappellen , J. Liu , Exploration 2024, 4, 20230018. 38939860 10.1002/EXP.20230018 PMC11189586

[14] L. G. Pinaeva , A. S. Noskov , Sci. Total Environ. 2024, 947, 174445. 38981547 10.1016/j.scitotenv.2024.174445

[15] Q. Chen , S. Wu , P. Zhang , X. M. Song , Z. Song , Green Chem. 2023, 25, 9146.

[16] B.‐N. T. Nguyen , J. Y. C. Lim , Trends Chem. 2024, 6, 100.

[17] Z. Sun , U. Glebe , H. Charan , A. Böker , C. Wu , Angew. Chem., Int. Ed. 2018, 57, 13810. 10.1002/anie.201806049 30141281

[18] S. Wang , L. Scandurra , R. Hübner , U. G. Nielsen , C. Wu , ChemCatChem 2023, 15, 202201229.

[19] R. Wei , G. von Haugwitz , L. Pfaff , J. Mican , C. P. S. Badenhorst , W. Liu , G. Weber , H. P. Austin , D. Bednar , J. Damborsky , U. T. Bornscheuer , ACS Catal. 2022, 12, 3382. 35368328 10.1021/acscatal.1c05856 PMC8939324

[20] Z. Sun , M. Cai , R. Hübner , M. B. Ansorge‐Schumacher , C. Wu , ChemSusChem 2020, 13, 6523. 33078882 10.1002/cssc.202002121

[21] Z. Sun , J. Jurica , R. Hübner , C. Wu , Catal. Sci. Technol. 2022, 12, 4811.

[22] Z. Gong , S. Gao , K. Lu , R. Hübner , C. Wu , Colloids Surf., A 2024, 682, 132922.

[23] Y. Wang , Q. Zhao , R. Haag , C. Wu , Angew. Chem., Int. Ed. 2022, 61, 202213974. 10.1002/anie.202213974 PMC10100074 36260531

[24] Z. Sun , C. Wu , Small 2024, 20, 2402208. 10.1002/smll.202402208 38716793

[25] F. Chang , C. M. Vis , W. Ciptonugroho , P. C. A. Bruijnincx , Green Chem. 2021, 23, 2575.

[26] L. Wei , M. Zhang , X. Zhang , H. Xin , H. Yang , ACS Sustainable Chem. Eng. 2016, 4, 6838.

[27] X. Guan , Z. Wan , T. Ngai , Aggregate 2023, 4, 300.

[28] W. Wang , Y. Yu , M. Wang , Y. Wang , S. Liu , J. Xu , Z. Sun , ACS Appl. Mater. Interfaces 2024, 16, 54799. 39315994 10.1021/acsami.4c10461

[29] Q. Zhao , M. B. Ansorge‐Schumacher , R. Haag , C. Wu , Bioresour. Technol. 2020, 295, 122221. 31615701 10.1016/j.biortech.2019.122221

[30] R. Röllig , C. Plikat , M. B. Ansorge‐Schumacher , Angew. Chem., Int. Ed. 2019, 58, 12960. 10.1002/anie.201907209 31218804

[31] H. Xie , W. Zhao , D. C. Ali , X. Zhang , Z. Wang , Catal. Sci. Technol. 2021, 11, 2816.

[32] K. Gao , W. C. Huang , H. Dong , J. Sun , H. Jiang , X. Mao , ACS Sustainable Chem. Eng. 2024, 12, 8061.

[33] S. Yu , D. Zhang , J. Jiang , Z. Cui , W. Xia , B. P. Binks , H. Yang , Green Chem. 2019, 21, 4062.

[34] Z. Chen , H. Ji , C. Zhao , E. Ju , J. Ren , X. Qu , Angew. Chem., Int. Ed. 2015, 54, 4904. 10.1002/anie.201412049 25706244

[35] M. Zhang , L. Wei , H. Chen , Z. Du , B. P. Binks , H. Yang , J. Am. Chem. Soc. 2016, 138, 10173. 27429173 10.1021/jacs.6b04265

[36] X. Wu , H. Karring , Z. Wang , C. Wu , Chem. Sci. 2025, 16, 4892. 39944123 10.1039/d4sc08063g PMC11811893

[37] Z. Sun , R. Hübner , J. Li , C. Wu , Nat. Commun. 2022, 13, 3142. 35668090 10.1038/s41467-022-30915-2 PMC9170730

[38] S. Wang , R. Hübner , H. Karring , V. F. Batista , C. Wu , Angew. Chem., Int. Ed. 2024, 64, 202416556. 10.1002/anie.202416556 39621003

[39] A. M. Bago Rodriguez , L. Schober , A. Hinzmann , H. Gröger , B. P. Binks , Angew. Chem., Int. Ed. 2021, 60, 1450. 10.1002/anie.202013171 PMC7839585 33119950

[40] C. Yin , X. Chen , H. Zhang , Y. Xue , H. Dong , X. Mao , Biotechnol. Adv. 2024, 72, 108338. 38460741 10.1016/j.biotechadv.2024.108338

[41] K. Zhang , A. H. Hamidian , A. Tubić , Y. Zhang , J. K. H. Fang , C. Wu , P. K. S. Lam , Environ. Pollut. 2021, 274, 116554. 33529891 10.1016/j.envpol.2021.116554

[42] H. H. Kim , B. J. Kim , Chem. Eng. J. 2024, 493, 152407.

[43] H. Firoozmand , D. Rousseau , Food Res. Int. 2016, 81, 66.

[44] P. Wongkongkatep , K. Manopwisedjaroen , P. Tiposoth , S. Archakunakorn , T. Pongtharangkul , M. Suphantharika , K. Honda , I. Hamachi , J. Wongkongkatep , Langmuir 2012, 28, 5729. 22443382 10.1021/la300660x

[45] Y. Teramura , H. Iwata , Soft Matter 2010, 6, 1081.

[46] N. Zhang , R. Hübner , Y. Wang , E. Zhang , Y. Zhou , S. Dong , C. Wu , ACS Appl. Nano Mater. 2018, 1, 6378.

[47] J. Ning , Z. Sun , R. Hübner , H. Karring , M. F. Ebbesen , M. Dimde , C. Wu , Nat. Catal. 2024, 7, 1404.

[48] X. Jiang , C. Yucel Falco , K. N. Dalby , H. Siegumfeldt , N. Arneborg , J. Risbo , Food Hydrocolloids 2019, 89, 224.

[49] D. C. Ali , X. Zhang , Z. Wang , Appl. Microbiol. Biotechnol. 2023, 107, 5843. 37466667 10.1007/s00253-023-12688-w

[50] Q. Zhao , M. B. Ansorge‐Schumacher , R. Haag , C. Wu , Chem. – Eur. J. 2018, 24, 10966. 29894011 10.1002/chem.201802458

[51] Z. Sun , Q. Zhao , R. Haag , C. Wu , ChemCatChem 2022, 14, 202101556.

[52] M. Zhang , Y. Bai , Z. Wang , X. Gao , W. Zhang , J. Li , W. Wu , H. Zhou , Q. Mei , Chem. Eng. J. 2024, 500, 156914.

[53] T. Suyama , Y. Tokiwa , Enzyme Microb. Technol. 1997, 20, 122.

[54] W. Yue , C. F. Yin , L. Sun , J. Zhang , Y. Xu , N. Y. Zhou , J. Hazard. Mater. 2021, 416, 125775. 33838511 10.1016/j.jhazmat.2021.125775

[55] E. V. Antonakou , D. S. Achilias , Waste Biomass Valorization 2013, 4, 9.

[56] V. Arumugam , R. Kanthapazham , D. A. Zherebtsov , K. Kalimuthu , P. Pichaimani , A. Muthukaruppan , Cellulose 2021, 28, 4847.

[57] I. Garikoé , B. Guel , I. Persson , Molecules 2022, 27, 4343. 35889216 10.3390/molecules27144343 PMC9316034

[58] M. C. R. Franssen , P. Steunenberg , E. L. Scott , H. Zuilhof , J. P. M. Sanders , Chem. Soc. Rev. 2013, 42, 6491. 23519171 10.1039/c3cs00004d

