# Supplementary material for: Pickering Emulsion Biocatalysis with Engineered Living Cells for Degrading Polycarbonate Plastics
Source: Small. 2025 May 24;21(29):2504376. doi: 10.1002/smll.202504376 (PMC12288795; doi:10.1002/smll.202504376)
Supplement: Supplementary file 1 — Supporting Information [file SMLL-21-2504376-s001.pdf]

NANO · MICRO
small

Supporting Information

for *Small*, DOI 10.1002/smll.202504376

Pickering Emulsion Biocatalysis with Engineered Living Cells for Degrading Polycarbonate Plastics

*Shan Wang, Zhimin Gong, René Hübner, Henrik Karring and Changzhu Wu**

Supporting Information

Pickering Emulsion Biocatalysis with Engineered Living Cells for Degrading Polycarbonate Plastics

Shan Wang,[a,1] Zhimin Gong,[a, b 1] René Hübner,[d] Henrik Karring,[e] Changzhu Wu[a, c,*]

[a] Department of Physics, Chemistry and Pharmacy, University of Southern Denmark, Campusvej 55, 5230 Odense, Denmark; Email: wu@sdu.dk

[b] School of Geographic Information and Tourism, Chuzhou University, Chuzhou, 239000, P.R. China

[c] Danish Institute for Advanced Study (DIAS), University of Southern Denmark, Campusvej 55, 5230 Odense, Denmark

[d] Helmholtz-Zentrum Dresden - Rossendorf (HZDR), Institute of Ion Beam Physics and Materials Research, Bautzner Landstrasse 400, 01328 Dresden, Germany

[e] Department of Green Technology, University of Southern Denmark, Campusvej 55, 5230 Odense, Denmark

Contents

1. Materials and instruments 3

2. Synthesis 3

    2.1 Synthesis of polyethyleneimine modified by alkyl chain 3

    2.2 Synthesis of PEI-alkyl modified with dye 4

3. PEI-alkyl coating on *Escherichia coli* (*E. coli*) cell surface 4

4. *Candida antarctica* Lipase B (CalB) expression 5

5. Green fluorescence protein (GFP) expression 5

6. Cell viability assay 5

7. Re-culturing cells and cell growth curve 5

8. Preparation of Pickering emulsion 6

9. Polycarbonate plastics degradation in Pickering emulsion 6

10. Results 7

11. Reference 11

1. Materials and instruments

All chemicals, unless otherwise specified, were purchased from commercial suppliers and used without further purification. Nonanoyl chloride, polyethyleneimine, 4,4'-isopropylidenediphenol (bisphenol A), and polycarbonate granules (nominal size: 3 mm) were obtained from Sigma-Aldrich.

Fourier-transform infrared spectroscopy (FTIR) measurements were done on a Cary 630 FTIR spectrometer (Agilent Technologies), scanning within the 648–4000 cm^{-1} range.

Proton nuclear magnetic resonance (^{1}H-NMR) spectra were acquired using Bruker AVANCE III and Jeol ECP 500 spectrometers, with tetramethylsilane (TMS) employed as the internal standard for chemical shift calibration.

Optical and fluorescence microscopy of cells were performed using a Nikon Ti2 Widefield microscope and Nikon Confocal A1 microscope, operated with NIS Elements software. Fluorescence channels were configured for excitation/emission in the 488 nm (FITC) and 561 nm (TRITC) ranges.

Scanning electron microscopy (SEM) analysis was carried out with an S-4800 microscope (Hitachi) at an accelerating voltage of 1 kV. Sample preparation for SEM followed the same procedure as described in our previous publication.[1]

Gas chromatography (GC) analysis was conducted on a SHIMADZU Nexis GC-2030 gas.

2. Synthesis

2.1 Synthesis of polyethyleneimine modified by alkyl chain

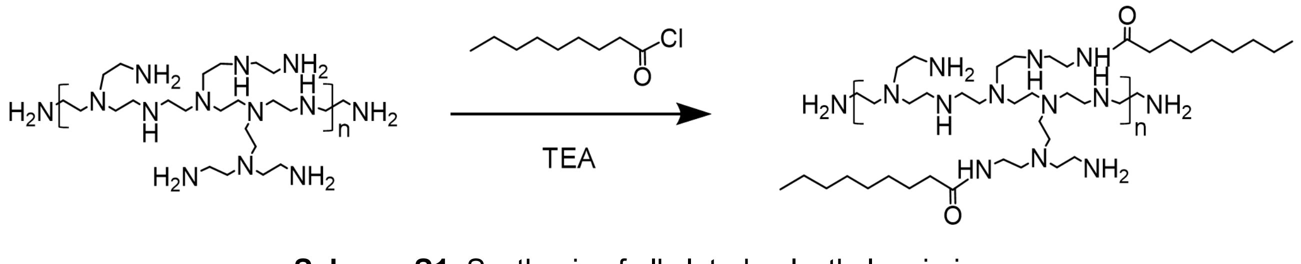

Scheme S1. Synthesis of alkylated polyethyleneimine.

In order to achieve the hydrophobic modification of cell surfaces, polyethyleneimine (PEI) was modified with alkyl chain to coat on the cell surfaces. Typically,[2] a PEI solution (50 wt.% in H_2O, 2.6 g, 3.34 mmol) was first dissolved in 100 mL DMSO. Then, nonanoyl chloride (0.98 g, 5.5 mmol) and triethylamine (0.59 mg, 6.0 mmol) were added inside in an ice bath. The mixture was stirred at

room temperature overnight. The supernatant was collected by centrifugation and dialysis against deionized water to get pure alkyl-chain-modified PEI, donated as PEI-alkyl.

2.2 Synthesis of PEI-alkyl modified with dye

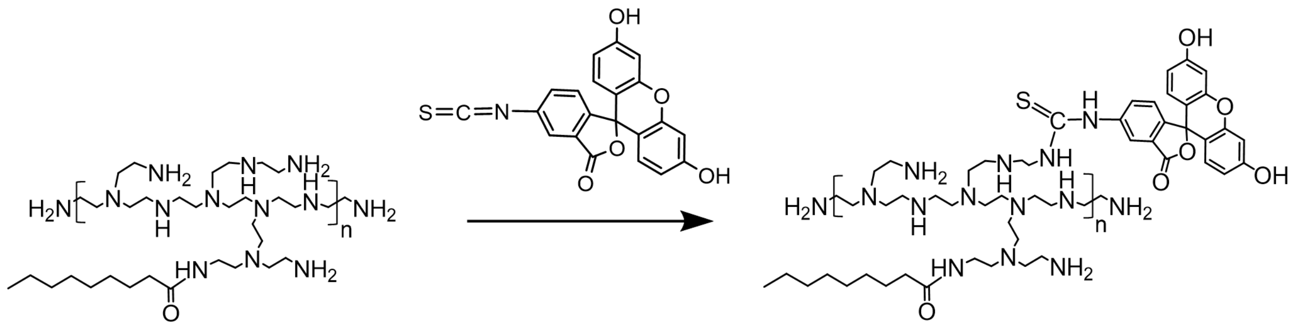

Scheme S2. Synthesis of PEI-alkyl labelled with dye.

In order to proof the PEI-alkyl coating on *E coli* cell surfaces, the PEI-alkyl was decorated with fluorescein isothiocyanate (FITC) as polymer dye to coat on the cells. Typically,[3] 100 mg PEI-alkyl were first dissolved in aqueous solution including 2 mg FITC. The mixture was stirred at room temperature overnight. The supernatant was collected by centrifugation and dialysis against deionized water to remove all free FITC. The obtained solution without free FTIC was freeze-dried to get a coloured solid, donated as PEI-alkyl-FITC. The final product was collected and directly used for coating onto the cells.

3. PEI-alkyl coating on *Escherichia coli* (*E. coli*) cell surface

Fresh *E. coli* cells were dispersed in 1 mL KPI buffer (pH 8.0, 100 mM) with OD_{600} of 2.0, and different amounts of PEI-alkyl (200 mg/mL, pH = 7) were added and mixed by shaking for 5 mins. After reaction, the PEI-alkyl-polymer-coated *E. coli* cells were collected by centrifugation, and then washed three times with KPi buffer (pH 8.0, 100 mM) to remove all free PEI-alkyl polymer. The obtained PEI-alkyl-coated cells (*E coli*@alkyl) were suspended in 1 mL KPI buffer (pH 8.0, 100 mM) and stored at 4 ℃ for further use.

The PEI-alkyl polymer with dye was coated onto cells using the same procedure, and the obtained *E. coli* cells with dye (*E coli*@alkyl-FITC) were confirmed using fluorescence microscopy.

4. *Candida antarctica* Lipase B (CalB) expression

CalB was expressed according to the reported method.[4] The *E. coli* BL21(DE3) strain carrying the plasmid pET22b_CalB-His was grown overnight in Luria-Bertani (LB) medium supplemented with 100 μg/mL ampicillin at 37 °C as a preculture. The main culture was then conducted in autoinduction medium at 20 °C overnight, following the protocol reported in our previous publication.[1] Cells overexpressing CalB were harvested by centrifugation at 5000 rpm and 4 °C, and the collected *E. coli* cells were subsequently washed with KPi buffer (100 mM, pH 8) and stored at 4 °C, for further use.

5. Green fluorescence protein (GFP) expression

GFP was expressed in *E. coli* BL21 (DE3) cells harbouring the plasmid pET-28b-GFP-His.[5] Precultures were prepared in LB medium supplemented with 50 μg/mL kanamycin and incubated at 37 °C with shaking at 220 rpm overnight. The preculture was then transferred to a main LB culture containing 50 μg/mL kanamycin. When the OD_{600} reached 0.8–1.0, GFP expression was induced by adding 0.5 mM isopropyl β-D-1-thiogalactopyranoside (IPTG), followed by incubation at 25 °C overnight. The bacterial pellets were harvested by centrifugation (4 °C, 6000 rpm, 15 minutes) and stored at 4 °C for further use.

6. Cell viability assay

Fresh native *E. coli* cells and PEI-alkyl-coated *E. coli* cells were initially suspended in KPI buffer (100 mM, pH 7.4). The cells were then stained with SYTO™ 9 and propidium iodide, respectively, by incubating them for 15 minutes at room temperature. After incubation, the cells were washed three times with KPi buffer (10 mM, pH 7.4) to remove any unbound dye. The stained cells were subsequently transferred for fluorescence microscopy analysis.

7. Re-culturing cells and cell growth curve

Fresh native *E. coli* cells and *E. coli*@alkyl cells were resuspended in 1 mL of LB medium containing 100 μg/mL ampicillin to an initial OD_{600} of 0.1. The resuspended cells were then incubated at 37 °C with shaking at 220 rpm. Cell growth was monitored over time by measuring OD_{600} values using UV-visible spectroscopy at designated intervals to generate growth curves.

8. Preparation of Pickering emulsion

The preparation process of Pickering emulsion involves mixing different ratios of water and toluene (the water : toluene ratio ranging from 2:8 to 8:2) in the presence of *E coli*@alkyl (OD_{600} = 2.0), followed by vortex shaking and sonication for 5 min to achieve a stable emulsion. The stability of the emulsion was monitored over time to observe the appearance of the emulsion, assessing the occurrence of phase separation. Fluorescence microscopy was employed to monitor the type of emulsion and to calculate the interface area between toluene and water. We observed that when the water-to-oil ratio was 5:5, the emulsion remained stable for over 196 hours, with no significant phase separation.

To get further information about the distribution of *E coli*@alkyl on the emulsion surface, the *E. coli* cells were overexpressed with GFP, and then conducted same coating procedure to get *E coli*(GFP)@alkyl. For the Pickering emulsion experiment, the *E coli*(GFP)@alkyl cells were immediately dispersed in 0.5 mL of Milli-Q water at an OD_{600} of 2.0, followed by the addition of 0.5 mL of toluene with vortex shaking and sonication to form a stable emulsion. The resulting emulsion was characterized using fluorescence microscopy to test the distribution of *E coli*(GFP)@alkyl in the Pickering emulsion.

9. Polycarbonate plastics degradation in Pickering emulsion

For polycarbonate plastics degradation in Pickering emulsion with CalB cell, *E. coli* cells overexpressing CalB were first coated using the same approach to obtain alkyl-chain-modified *E. coli* cells (*E. coli*(CalB)@Alkyl), which were subsequently redispersed in 0.5 mL of KPi buffer (pH 7, 10 mM). Polycarbonate plastics was swollen in 0.5 mL of toluene until saturation was reached. The KPi buffer containing *E. coli*(CalB)@alkyl (OD_{600} = 2.0) was then added to the toluene solution, followed by vortex shaking and sonication to form a stable emulsion. The reaction was carried out without stirring, and the reaction was monitored at different time intervals using gas chromatography (GC) to quantify the final degradation product, bisphenol A.

The control experiment was conducted under identical conditions using the same amount of native CalB cells in a two-phase reaction system. Optimization experiments of PC degradation at different temperatures (25 °C, 35 °C, and 45 °C) and various pH (5, 7, and 9) values were conducted following the same protocol.

E. coli(CalB)@alkyl was centrifuged and washed with KPi buffer (pH 7, 10 mM) before being used for the study of its recyclability.

10. Results

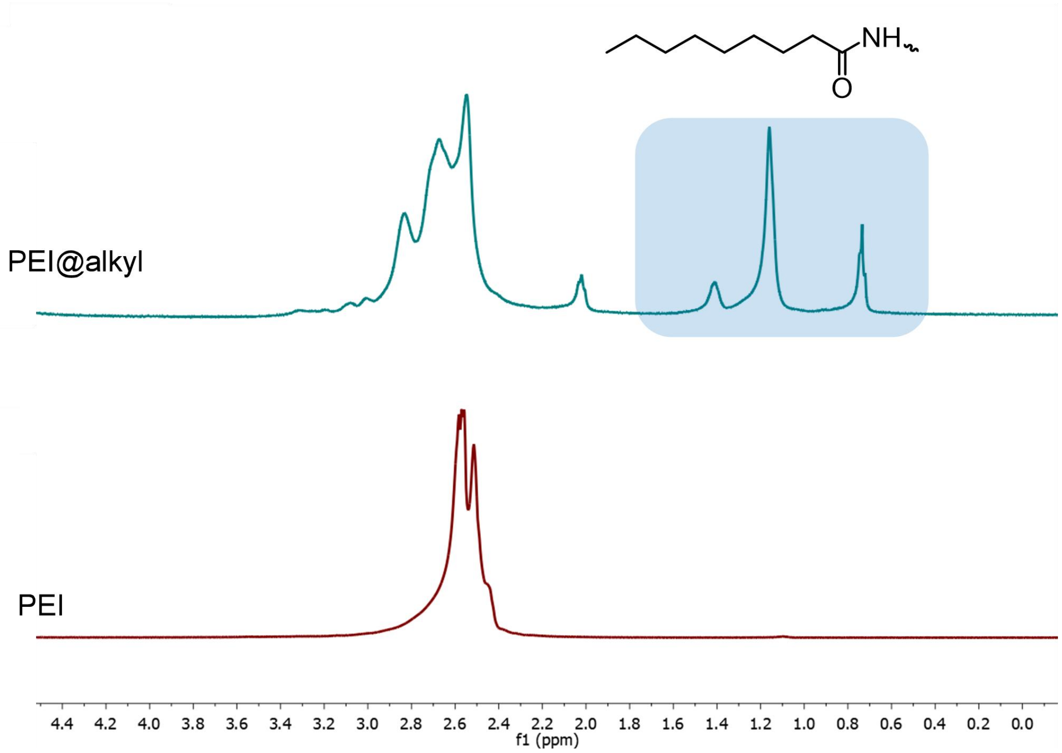

Fig.S1 ^{1}H-NMR spectra of PEI and PEI@alkyl.

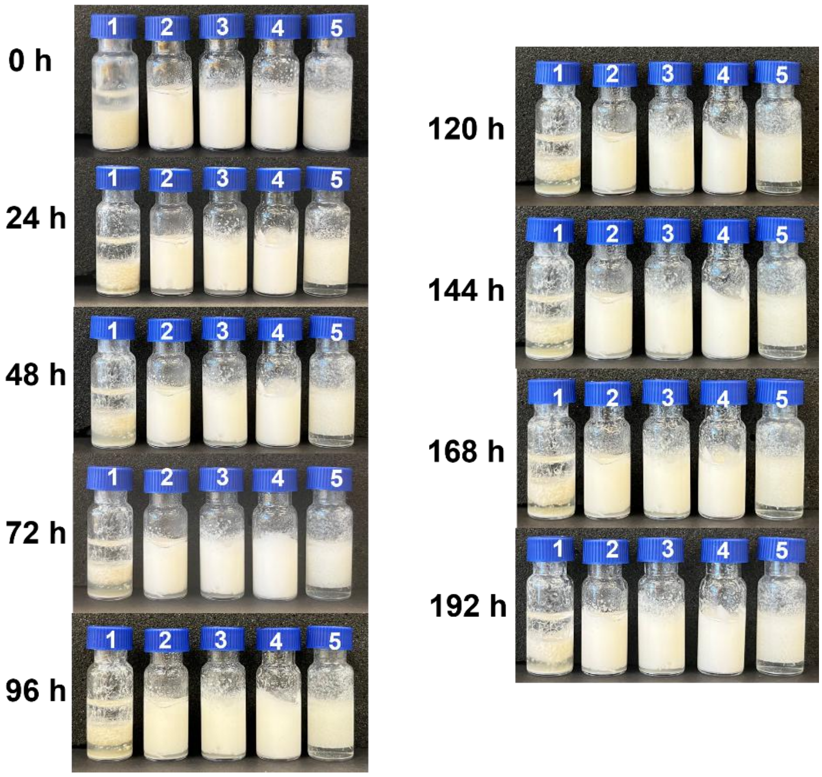

Fig.S2 Digital images of Pickering emulsions prepared using different concentration of PEI-alkyl polymers to coat *E. coli* cells for stabilizing emulsion, corresponding to 0 (**1**), 10 (**2**), 20 (**3**), 30 (**4**), and 40 mg/mL (**5**) of PEI-alkyl, respectively. The ratio of water-to-oil was set at 5:5, and the OD_{600} of *E coli*@alkyl was 2.0. Digital images of the bottles were taken at different times after preparation (0, 24, 48, 72, 96, 120, 144, 168, and 192 hours).

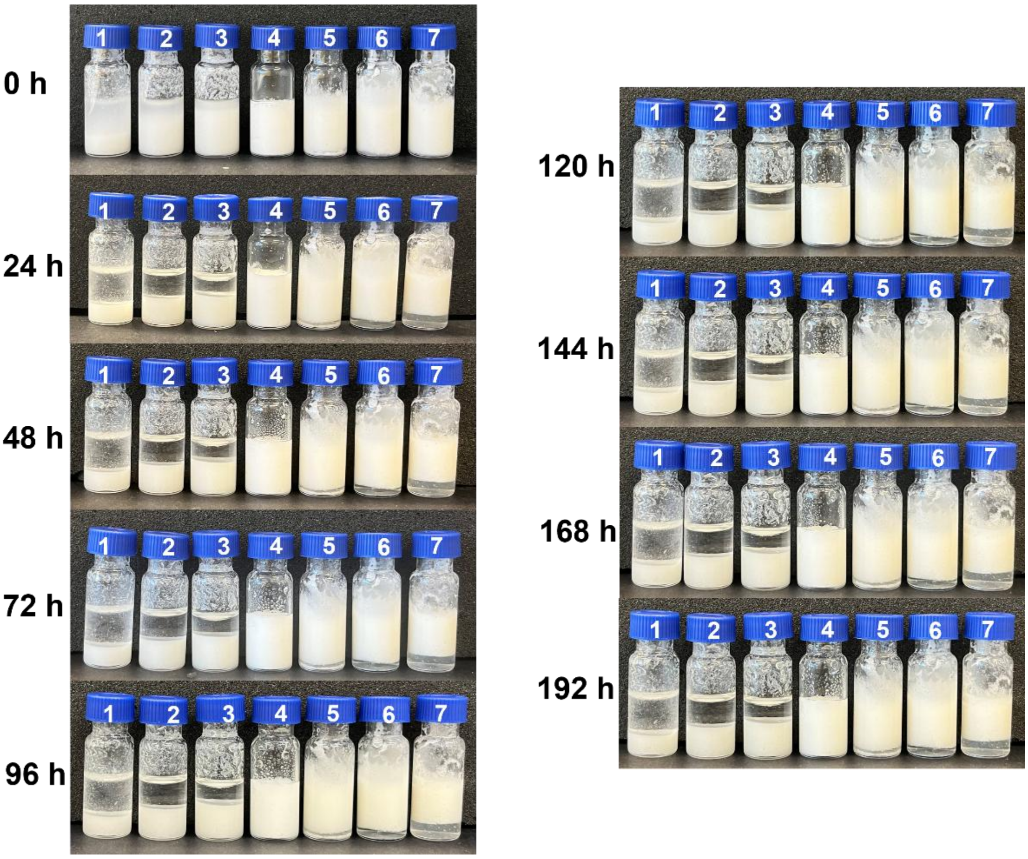

Fig.S3 Digital images of Pickering emulsions with *E.coli*@alkyl for different water-to-oil ratios. V_{H2O}:$V_{Toluene}$ = 2:8 (**1**), V_{H2O}:$V_{Toluene}$ = 3:7 (**2**), V_{H2O}:$V_{Toluene}$ = 4:6 (**3**), V_{H2O}:$V_{Toluene}$ = 5:5 (**4**), V_{H2O}:$V_{Toluene}$ = 6:4 (**5**), V_{H2O}:$V_{Toluene}$ = 7:3 (**6**), V_{H2O}:$V_{Toluene}$ = 8:2 (**7**). 100 µL of PEI-alkyl solution (200 mg/mL, pH 7) were used to obtain *E.coli*@alkyl-stabilized Pickering emulsion.

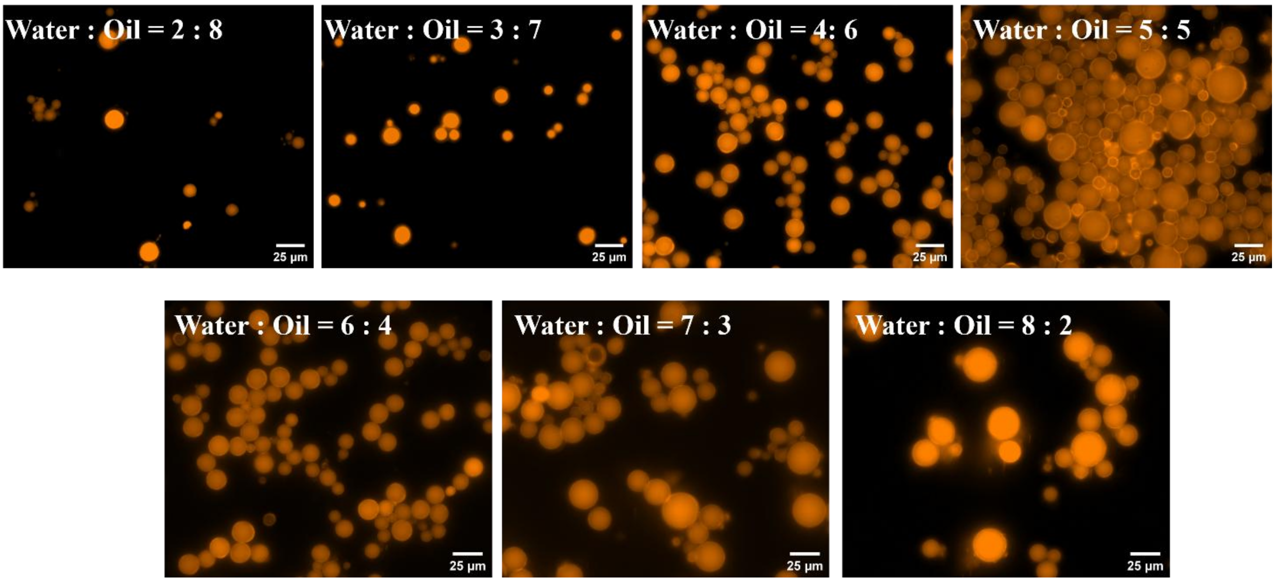

Fig.S4 Fluorescence microscopy images of emulsions with different water-to-oil ratios.

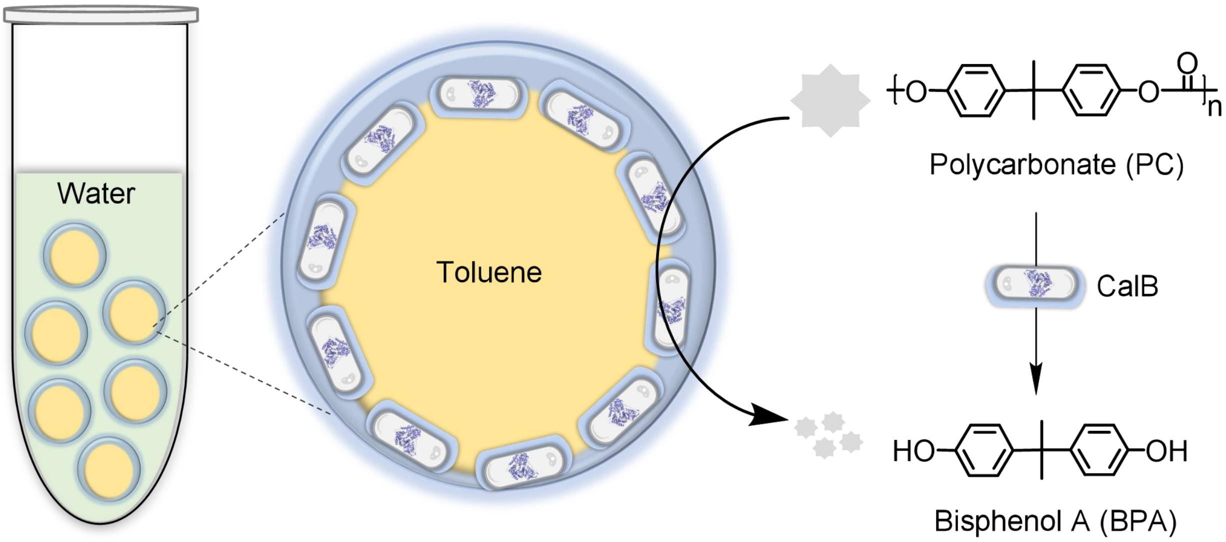

Fig.S5 Schematic demonstration for PC degradation in *E. coli*(CalB)@alkyl-stabilized Pickering emulsion.

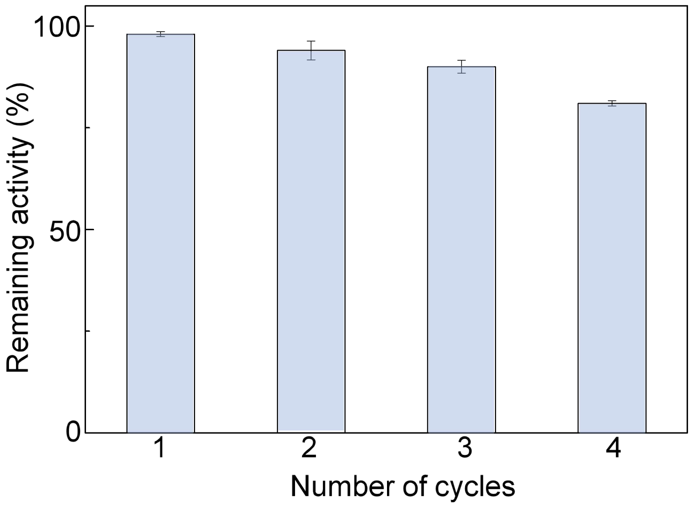

Fig.S6 The reusability of *E. coli*(CalB)@alkyl for PC biodegradation in Pickering emulsion. Data are expressed as mean ± standard deviation (s.d.), calculated from three independent replicates (n = 3); error bars indicate the s.d.

11. Reference

[1] Z. Sun, R. Hübner, J. Li, C. Wu, *Nat. Commun.* **2022**, *13*, 3142.

[2] Z. Sun, M. Cai, R. Hübner, M. B. Ansorge-Schumacher, C. Wu, *ChemSusChem* **2020**, *13*, 6523-6527.

[3] N. Zhang, R. Hübner, Y. Wang, E. Zhang, Y. Zhou, S. Dong, C. Wu, *ACS Appl. Nano Mater.* **2018**, *1*, 6378-6386.

[4] F. W. Studier, *Protein Expression Purif.* **2005**, *41*, 207-234.

[5] N. Zhang, Z. Sun, C. Wu, *ACS Catal.* **2022**, *12*, 4777-4783.
